# Monolithically integrated stretchable photonics

**DOI:** 10.1038/lsa.2017.138

**Published:** 2018-02-09

**Authors:** Lan Li, Hongtao Lin, Shutao Qiao, Yi-Zhong Huang, Jun-Ying Li, Jérôme Michon, Tian Gu, Carlos Alosno-Ramos, Laurent Vivien, Anupama Yadav, Kathleen Richardson, Nanshu Lu, Juejun Hu

**Affiliations:** 1 Department of Materials Science & Engineering, Massachusetts Institute of Technology, Cambridge, Massachusetts, USA; 2 Department of Aerospace Engineering and Engineering Mechanics, University of Texas at Austin, Austin, Texas, USA; 3 Department of Electronic Engineering, Xiamen University, Xiamen, China; 4 Key Laboratory of Optoelectronic Technology & System, Education Ministry of China, College of Opto-Electronic Engineering, Chongqing University, Chongqing, China; 5 Centre for Nanoscience and Nanotechnology, CNRS, Univ. Paris-Sud, Universite Paris-Saclay, Orsay, C2N—Orsay, France; 6 The College of Optics & Photonics, University of Central Florida, Orlando, Florida, USA

**Keywords:** chalcogenide glass, integrated photonics, optical resonator, strain-optical coupling, stretchable photonics

## Abstract

Mechanically stretchable photonics provides a new geometric degree of freedom for photonic system design and foresees applications ranging from artificial skins to soft wearable electronics. Here we describe the design and experimental realization of the first single-mode stretchable photonic devices. These devices, made of chalcogenide glass and epoxy polymer materials, are monolithically integrated on elastomer substrates. To impart mechanical stretching capability to devices built using these intrinsically brittle materials, our design strategy involves local substrate stiffening to minimize shape deformation of critical photonic components, and interconnecting optical waveguides assuming a meandering Euler spiral geometry to mitigate radiative optical loss. Devices fabricated following such design can sustain 41% nominal tensile strain and 3000 stretching cycles without measurable degradation in optical performance. In addition, we present a rigorous analytical model to quantitatively predict stress-optical coupling behavior in waveguide devices of arbitrary geometry without using a single fitting parameter.

## Introduction

In recent years, the increasing penetration of flexible devices into the consumer products market has led to a surge of interest in mechanically flexible photonics^1, 2^. In addition to being an essential component in consumer electronics, flexible photonics is now enabling a plethora of emerging applications including board-level optical interconnects^3, 4, 5, 6, 7, 8^, optomechanical tuning^9, 10, 11^, epidermal monitoring^12^, strain sensing^13^, and conformal photonics^14, 15^. While our earlier work has led to foldable photonic devices with record optical performance and extraordinary mechanical ruggedness^16, 17, 18^, it is often mandated in these applications that the devices are not only bendable, but also stretchable. For instance, human skin is a soft elastic material with up to 20% stretchability and perfect reversibility^19^, which demands epidermal devices to exhibit commensurate deformation capability. Stretching capability is also indispensable for wrinkle-free conformal integration on curvilinear surfaces. Previously reported stretchable integrated photonics have been entirely made of elastomer materials^20, 21^, which severely limits the available material options. Furthermore, the elastomer waveguides are heavily multimode, which poses a major constraint for many applications. While hybrid transfer based techniques can be applied to produce stretchable structures comprising isolated dielectric nanorods embedded inside an elastomer matrix^22, 23, 24^, this particular geometry is incompatible with most integrated photonic devices.

In this paper, we present the design and the first experimental demonstration of single-mode stretchable integrated photonic devices fabricated using chalcogenide glass (ChG) and epoxy polymer. Our material selections allow photonic components with both high and low index contrasts to be seamlessly integrated in the same device platform. This unique feature combines the best of both worlds: for example, we can take advantage of the reduced propagation loss in low-index-contrast optics while leveraging high-index-contrast (HIC) elements to attain tight optical confinement and superior diffraction efficiency. The material choices further enable monolithic photonic integration on elastomeric substrates, formerly considered a challenging task due to the gigantic coefficient of thermal expansion (CTE) of elastomers^25, 26, 27, 28^, which is one to two orders of magnitude larger than those of inorganic semiconductor and dielectric materials.

Both the glass and epoxy polymer, however, are intrinsically brittle. To impart mechanical stretchability to devices made of these materials, we have developed a mechanical design where functional optical components are located on isolated stiff ‘islands’ and interconnected through optical waveguides with a serpentine shape. Local substrate stiffening at the islands suppresses strain exerted on the optical components^29^. Analogous to helical springs, the serpentine waveguide geometry can accommodate large elongation without fracture. We note that while similar meandering metal wire designs have already been well formulated in the context of stretchable electronic circuits^30, 31, 32, 33^, such layouts have to be judiciously re-engineered to mitigate excessive radiative optical losses when adapted to integrated optical waveguides.

These mechanical design principles are experimentally implemented to realize highly stretchable photonic circuits and quantitatively validated through finite element mechanical models coupled with a rigorous strain-optical coupling theory. Unlike previously formulated strain-optical coupling analyses which either apply only to limited cases of specific waveguide geometries^34, 35^ or involve parameters that cannot be straightforwardly evaluated and hence are descriptive in nature^13^, our new approach enables quantitative prediction of strain-induced drift of device optical characteristics without a single fitting parameter.

The design rationale, theoretical framework and integration routes outlined above are elaborated in the following sections. They are also generically applicable to mechanically flexible optical systems made from a wide cross-section of materials to meet diverse needs in various application scenarios.

## Materials and methods

### Stretchable device fabrication

Device fabrication was performed at the MIT Microsystems Technology Laboratories and the Harvard Center for Nanoscale Systems. The handler substrates are bare silicon wafers cleaved into 1’ by 1’ square pieces (Silicon Quest International). The following layers were then sequentially coated on the handler substrates: polydimethylsiloxane (PDMS) (3 μm thick, Dow Corning Sylgard 184 elastomer with a 10:1 monomer/curing agent mixing ratio and diluted in hexane), SU-8 (0.8 μm thick, Microchem SU-8 2000.5), and Ge_23_Sb_7_S_70_ glass (450 nm thick). The PDMS and SU-8 layers were formed via spin coating. Prior to PDMS coating, the substrates were silanized to facilitate structure delamination upon completion of fabrication. The substrates were also briefly treated in oxygen plasma before SU-8 coating to promote layer adhesion. The Ge_23_Sb_7_S_70_ glass film was deposited via thermal evaporation using a custom-designed system (PVD Products, Inc.), during which the substrate was held near room temperature^36, 37^. The deposition rate was monitored in real-time using a quartz crystal microbalance and was stabilized at 20Å s^−1^. The glass film was subsequently patterned on an Elionix ELS-F125 electron beam lithography system using Microchem SU-8 2000.2 as the electron beam resist followed by fluorine plasma etching^38^. The etched glass photonic devices were encapsulated in a second laminate of SU-8 epoxy (Microchem SU-8 2002), which also serves as an electron beam resist and an etch mask to define the serpentine structures in the SU-8 layers. Finally, a second PDMS layer of about 100 μm in thickness (10:1 monomer/curing agent mixing ratio and without hexane dilution) was spin coated and the entire structure was delaminated from the handler using a water-soluble tape (WST-1, kaptontape.com), which was later removed by rinsing in diluted HCl solution. The fabricated devices were flood exposed under a halogen lamp before optical testing to nullify the glass film’s photosensitivity.

### Calibration sample fabrication

A 2.8-μm-thick SU-8 film (Microchem SU-8 2002) was first coated on a hander silicon wafer with 300-nm thermal oxide (Silicon Quest International). A 450-nm-thick Ge_23_Sb_7_S_70_ glass film was then thermally evaporated onto the substrate. Fabry-Perot cavities were subsequently patterned using electron beam lithography and capped with another 40-μm-thick SU-8 epoxy layer (Microchem SU-8 2025) before delaminated from the handler substrate using a Kapton tape to form freestanding, bendable membranes^16^.

### Device characterization

**Figure 1:**
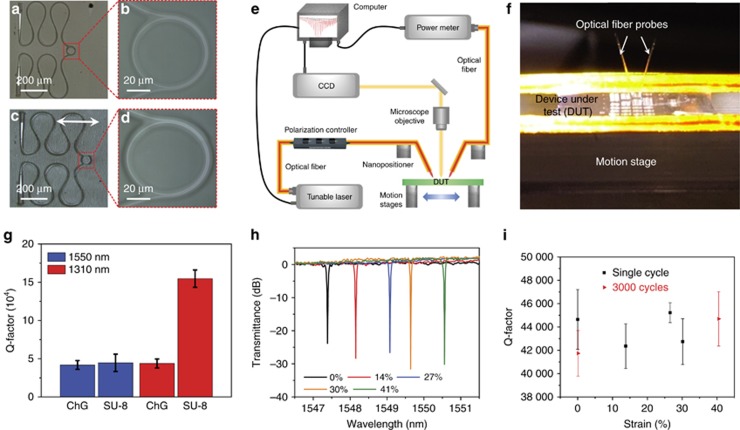
(**a**–**d**) Top-view micrographs of a stretchable device (**a**, **b**) in its undeformed state and (**c**, **d**) at 36% nominal tensile strain; the arrow in **c** indicates the stretching direction; (**e**) schematic diagram of the experimental characterization setup; (**f**) a photo of a stretched device under test; (**g**) measured TE-polarization Q-factors of ChG/SU-8 (labeled as ‘ChG’) and SU-8/PDMS (labeled as ‘SU-8’) resonator devices at 1310 and 1550 nm wavelengths; (**h**) normalized optical transmittance spectra of a ChG/SU-8 stretchable resonator at different nominal strain levels; (**i**) Q-factors of ChG/SU-8 resonator devices before and after 3000 stretching cycles at 41% nominal strain. The error bars indicate standard deviations of resonant peaks between the wavelength ranges of 1540–1570 nm. DUT, device under test.

Figure 1e schematically illustrates the optomechanical measurement setup used to characterize the stretchable photonic devices. The two ends of the fabricated devices in the form of stretchable membranes were mounted to a couple of linear motion stages. Nominal tensile strain applied on the membrane was controlled by changing the distance between the two motion stages. A pair of single-mode fiber probes with cleaved end facets were used to couple light from an external cavity tunable laser (Luna Technologies) to the devices via grating couplers and back to an optical vector analyzer for spectrally resolved transmittance measurement. Figure 1f shows a stretched device under test.

### Finite element modeling

**Figure 2:**
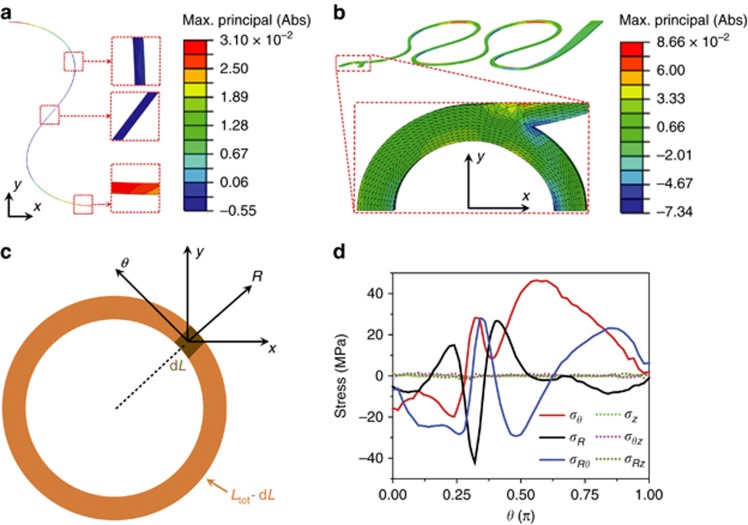
Micro-mechanical FEM simulations: (**a**) strain distribution in an Euler-spiral-shaped ChG waveguide: the insets plot the strain profiles at high-symmetry points of the Euler spiral structure; (**b**) strain field in the stretchable device structure shown in Figure 1a; (**c**) schematic top-view layout of a ChG micro-ring resonator; (**d**) stress components along the azimuth of a ChG micro-ring resonator in the stretchable device structure of Figure 1a. The strain components are defined with respect to the coordinate systems illustrated in **c** and *z* is the out-of-plane direction. All the simulation results correspond to the case of 41% nominal tensile strain. Max., maximum.

To obtain the strain field of the stretchable photonics, we have conducted numerical simulations with finite element method (FEM) using the commercial software ABAQUS v6.14. The elastic properties of materials used in the simulations are either quoted from the manufacturer’s specifications (for SU-8) or experimentally measured using dynamic mechanical analysis (for PDMS) or the ultrasound pulse-echo technique (for Ge_23_Sb_7_S_70_). The stretchable photonic devices embedded in a PDMS matrix were modeled. Taking geometric symmetry into account, only half of the device and the matrix were needed in the simulation, which reduces the computational cost. Moreover, since SU-8 and Ge_23_Sb_7_S_70_ glass have similar mechanical properties, the photonic device was modeled as a device made of SU-8 only. As discussed in Results and Discussion section, SU-8 is much stiffer than the PDMS matrix, therefore, upon a far-field load, strains in the PDMS matrix are expected to be much larger compared to that of the photonic device. Thus, the device material (SU-8) was considered linear elastic whereas PDMS was modeled as incompressible Neo-Hookean material. C3D4 and C3D4H elements were used to mesh the photonic device and the PDMS matrix, respectively. To elucidate the superior mechanical robustness of the device, a 41% uniaxial tensile strain, which is far beyond the failure strain of the device materials, was applied to the matrix in the far field. At equilibrium status, strains in the device was computed and shown in Figure 2.

## Results and discussion

### Design considerations and fabrication protocols

**Figure 3:**
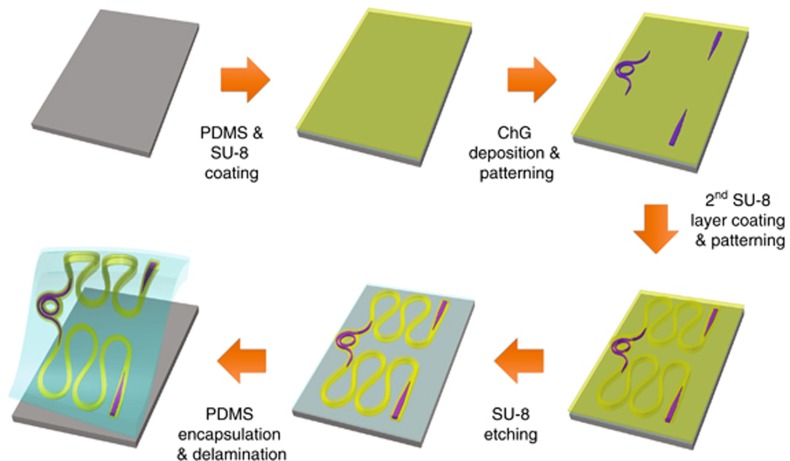
Schematic fabrication process flow of the stretchable photonic devices.

The fabrication flow of stretchable photonic devices is schematically illustrated in Figure 3, and details of the process are furnished in Materials and Methods section. In the following we summarize the key fabrication steps and our rationale for material choices and device design.

The process starts with a handler silicon wafer on which a polydimethylsiloxane (PDMS) elastomer layer and an SU-8 epoxy film are sequentially coated. The PDMS layer acts as the stretchable substrate; and the SU-8 epoxy film fulfills several functions. First, it has an intermediate CTE of 52 ppm °C^−1^, which lies between those of the Ge_23_Sb_7_S_70_ chalcogenide glass (17.1 ppm °C^−1^) and PDMS (310 ppm °C^−1^)^39^. In effect, the epoxy film serves as a thermal stress release layer to bridge the large CTE mismatch between the glass and PDMS. The stress suppression effect is evident from our own fabrication results: in the absence of the SU-8 layer, ChG films deposited and patterned directly on PDMS suffer from severe damage, whereas no cracks were visible under optical microscope inspection in structures containing the SU-8 buffer layer (Supplementary Information Section I). In addition, SU-8 is mechanically rugged with a Young’s modulus of 2.0 GPa, close to that of Ge_23_Sb_7_S_70_ glass (16.4 GPa) and much higher than that of PDMS (2.6 MPa). Therefore, lithographically patterned SU-8 pads provide the locally stiff ‘islands’ on which the critical optical components locate. Finally, SU-8 is optically transparent and has a refractive index of 1.57 (measured at 1310 and 1550 nm telecommunication bands), slightly higher than that of PDMS (1.40). SU-8 can thus function as a low-index-contrast waveguide core for light transmission.

We recognize that the combination of SU-8 and PDMS, while suitable for low loss waveguiding, does not offer sufficient index contrast for optical functions such as diffractive coupling, tight waveguide bends, or a complete photonic bandgap. A Ge_23_Sb_7_S_70_ chalcogenide glass film with a refractive index of 2.22 (measured at 1550 nm wavelength) is subsequently deposited and lithographically patterned to form HIC structures (for example, grating couplers). In these HIC components, the SU-8 epoxy is used simultaneously as the optical cladding and as mechanical support to locally stiffen the substrate and minimize undesired geometric deformations as well as ensuing strain-induced operation wavelength drift. We further note that while here we chose the specific glass composition of Ge_23_Sb_7_S_70_, monolithic integration of ChG photonics on epoxy films had been previously validated in several ChG composition groups^16^, thereby offering significant flexibility for optical design and device engineering. To facilitate optical coupling between the low-index-contrast SU-8/PDMS waveguides and HIC ChG/SU-8 elements, we designed and fabricated adiabatic mode transformers with a low insertion loss of (0.4±0.2) dB per coupler (Supplementary Information Section II).

In the next step, a second SU-8 epoxy layer is spin-coated and patterned to serve both as the top optical cladding for the ChG devices and as an etch mask to define serpentine waveguide structures in the bottom SU-8 layer. Here the advantage of using SU-8 as the etch mask is that itself is also an electron beam resist and thus can be readily patterned via electron beam writing. The serpentine waveguide layout is designed to enhance mechanical stretchability of the structure. In stretchable electronics, a horseshoe design consisting of piecewise circular segments have been optimized for maximizing stretchability of metal wires^40^. When applied to optical waveguides, the design nevertheless results in excessive radiative optical loss due to the abrupt curvature change at the junctions between the circular arcs. To mitigate such scattering losses, waveguide structures with continuously varying curvature following Bezier curve or Euler spiral geometries have been adopted^41, 42^. We therefore implemented an Euler spiral design in our serpentine waveguide layout, which allows up to 64% propagation loss reduction compared with the traditional horseshoe structures while contributing to large stretchability (Supplementary Information Section III).

Finally, the fabricated devices are encapsulated by a second PDMS layer and delaminated from the handler wafer in the form of freestanding stretchable membranes for optical and mechanical characterizations.

### Optical and mechanical characterizations

Figure 1a and 1b shows optical micrographs of a sample in its undeformed state. As shown in the figure, a typical device under test comprises a micro-ring resonator connected to two grating couplers through meandering waveguides assuming an Euler spiral geometry. The grating couplers and the micro-ring resonator are encapsulated in SU-8 islands to minimize shape distortion during stretching. The grating couplers are optimized for TE polarized light and fabricated using Ge_23_Sb_7_S_70_ glass embedded in SU-8, whereas two types of micro-ring resonators made of SU-8/PDMS (core/cladding) and ChG/SU-8, respectively, are fabricated and tested.

During optical testing, TE-polarized laser light was coupled into the stretchable devices via ChG grating couplers embedded in the SU-8 layer. The sample was mounted on a pair of linear translational stages which precisely controlled the nominal mechanical strain (defined as the fractional elongation of the entire sample along the stretching direction) applied on the sample (Figure 1e and Materials and Methods section). Figure 1g compares the measured intrinsic quality factors (Q’s) of the SU-8/PDMS and ChG/SU-8 resonators at 1310 and 1550 nm wavelengths. Our results obtained in the SU-8/PDMS devices are comparable to the best previously reported Q values in SU-8 resonators on rigid substrates^43, 44, 45, 46^. The lower Q-factor of SU-8/PDMS rings at 1550 nm is attributed to C-H bond overtone absorption in SU-8^43^.

For comparison, optical performance of the devices was also monitored as the samples underwent tensile strain. Figure 1c and 1d shows optical microscope images of a device at 36% nominal strain. While shape change of the serpentine waveguides is apparent, no cracks or defects were visually discernable during or after repeated stretching (Supplementary Information Section IV). Figure 1h presents the transmission spectra of a ChG/SU-8 resonator under different strains. The waveguide propagation loss remained unchanged at different strain states. We note that the resonant peak red shifts with increasing strain: such strain-optical coupling behavior is quantitatively accounted for using a model detailed in a succeeding section. Figure 1i plots the measured intrinsic Q-factors of the resonator. No measurable change was observed after 3000 stretching cycles at 41% nominal strain, which attests to the exceptional mechanical ruggedness of the device.

### Mechanical modeling

We constructed finite element method (FEM) models to elucidate the superior mechanical robustness of the device despite the intrinsic brittleness of its constituent materials. Details of the FEM modeling is described in Materials and Methods section. Figure 2a depicts the strain distribution in a half-period Euler spiral structure. With 41% overall structure elongation, the maximum strain in the Euler spiral structure is only 3%: this 14-fold strain reduction clearly signifies the strain suppression efficacy of the design. To evaluate the effect of local substrate stiffening, we also modeled the stretching behavior of the device layout shown in Figure 1a. Figure 2b maps the strain fields in the SU-8 ‘islands’ and Figure 2d plots the local stresses along the ChG micro-ring embedded inside the SU-8 supporting structure, both calculated at 41% elongation of the entire device. The stresses along the micro-ring correspond to an average strain of merely 0.77%, 55 times lower than the nominal strain exerted on the structure. We therefore conclude that local substrate stiffening is highly effective in stabilizing the operation of photonic devices whose optical characteristics are sensitive to geometric deformation.

### Strain-optical coupling in flexible photonic devices: a predictive model

As shown in Figure 1h, the resonant peak of the micro-ring device red shifts with increasing tensile strain. Similar strain-optical coupling response has also been reported and theoretically analyzed in several prior publications^13, 16, 47, 48, 49^. According to the theoretical models^13, 48^, there are three effects contributing to the observed strain-optical coupling: strain-induced waveguide effective index and length changes due to dimensional variations, and photoelastic effect which modifies the refractive indices of waveguide core and cladding materials. While the physics of such coupling is relatively well-established, these models suffer from a major drawback: they are unable to provide quantitative predictions on the strain-optical coupling characteristics and can only be used to fit experimentally measured data retrospectively. The key challenge resides in accurate assessment of photoelastic effects in thin film materials: the sub-micron thickness of optical thin films renders traditional phase retardation analysis techniques^50^ unreliable for quantifying the weak photoelastic interactions in most inorganic materials (for example, the stress-optical coefficient is in the order of 10^−12^ Pa^−1^ in inorganic glasses, one to two orders of magnitude smaller than the typical values in polymers). A technique involving temperature-dependent prism coupling measurements performed on the same film deposited on multiple different substrate materials was devised^51^, although it has poor accuracy (due to the limited refractive index resolution of prism coupling at>10^−4^) and mandates prior knowledge about the film and substrates (including elastic moduli, Poisson ratio, CTE and so on) which may not be readily available. In addition to the difficulties associated with extracting photoelastic parameters, these early models also only consider the oversimplified case of uniform strain and neglect the tensorial nature of strain and stress.

Here we developed a tensorial stress-optical coupling model generically applicable to arbitrary spatially varying stress profiles and waveguide geometries. The theoretical derivation is presented in Supplementary Information Section V. For the sake of brevity, we directly quote the general result of resonance wavelength shift caused by applied stress *σ*:


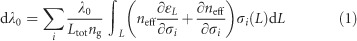


where *λ*_0_ represents the resonant wavelength (in free space), *L*_tot_ is the resonator length, *n*_g_ and *n*_eff_ are the modal group and effective indices respectively, *ε*_*L*_ denotes axial strain along the waveguide, and the contour integral is carried out along the resonator waveguide path. In Equation (1), the summation is performed over all normal stress tensor components, as we have proven that shear stress components have a negligible impact on waveguiding properties of photonic devices (Supplementary Information Section VI). Here we formulate the problem in terms of stress instead of strain because normal stress in the surface-normal direction vanishes in flexible photonic systems taking the form of a freestanding membrane, which is evidenced by the FEM results in Figure 2d showing that *σ*_*z*_ is negligible compared with *σ*_*R*_ or *σ*_*θ*_. Therefore, for a circularly symmetric micro-ring resonator the summation in Equation (1) reduces to:


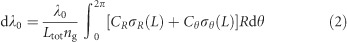


where *R* is the resonator radius in the absence of perturbation and the two stress-optical coupling coefficients *C* are defined as:


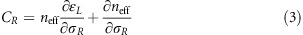


and


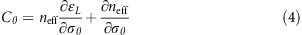


which represent the stress-optical coupling strength in the radial (*R*) and circumferential (*θ*) directions of the resonator (Figure 2c), respectively.

**Figure 4:**
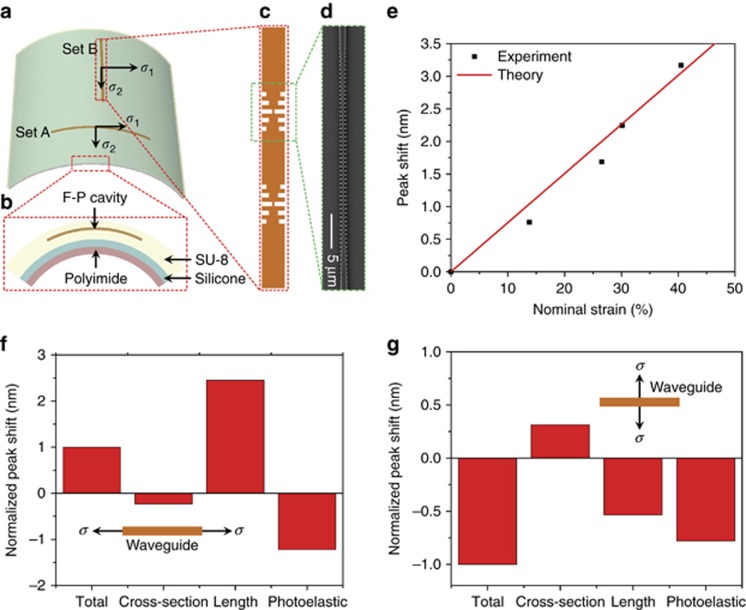
(**a**) Schematic layout of the calibration sample; (**b**) cross-sectional structure of the calibration sample; (**c**) schematic illustration of the F-P cavity design, which consists of a straight waveguide segment situated between a pair of Bragg grating reflectors; (**d**) top-view SEM micrograph of the waveguide Bragg grating reflector; (**e**) comparison of the experimentally determined strain-induced resonance shifts according to Figure 1h (points) and our theoretical prediction (solid line). Note that the theory does not involve any fitting parameters from the stretchable device measurement; (**f**, **g**) calculated relative contributions of photoleastic effects, waveguide cross-sectional modification, and waveguide length change to overall strain-induced resonance shift (normalized as unity) when in-plane uniaxial tensile stress is applied **f** along the resonator waveguide and **g** perpendicular to the waveguide. The positive/negative signs indicate red/blue shifts, respectively. Insets illustrate the stressed waveguide configurations.

To quantify the two stress-optical coupling coefficients, we employed a calibration sample encompassing two sets of Fabry-Perot (F-P) waveguide Bragg cavities oriented in orthogonal directions, as illustrated in Figure 4a. Each set consists of F-P cavities of varying lengths to eliminate stress-optical coupling contributions from the Bragg reflectors, and the waveguide sections in the cavities have a uniform cross-sectional geometry identical to that of the micro-ring resonator. The sample was bent to a series of radii and the corresponding resonance drifts of the two sets of cavities were monitored. As discussed in Supplementary Information Section VII, such stress-induced resonance shifts in the two orthogonal sets of devices allow us to independently evaluate the two stress-optical coupling constants *C*_*R*_ and *C*_*θ*_. The measured *C*_*R*_ and *C*_*θ*_ values and spatially varying stress field along the micro-ring simulated using FEM (Figure 2d) were then substituted into Equation (2) to predict the resonance wavelength shift in stretchable devices shown in Figure 1h. Figure 4e compares the theoretical predictions made using Equation (2) (solid line) and the measured resonance shift from Figure 1h (dots), and the excellent agreement substantiates our stress-optical coupling theory. We note that the theoretical prediction in Figure 4e was made solely based on either experimentally measured parameters on the calibration sample or FEM simulation results rather than data from the stretchable device under investigation, and therefore our model can indeed be applied to predict strain-optical coupling behavior in flexible photonic devices.

Our approach also offers an accurate route to quantify photoelastic effects in the waveguide materials (refer to Supplementary Information Section VIII for detailed analysis procedures). Figure 4f and 4g delineates the relative contributions of photoleastic effects, waveguide cross-sectional geometry modification, and waveguide length change to overall resonance shift (normalized as unity) when in-plane uniaxial tensile stress is applied along the resonator waveguide (Figure 4f) and perpendicular to the waveguide (Figure 4g). Notably, in the latter case tensile stress produces blue detuning of the cavity resonance because of Poisson’s effect and refractive index decrease due to photoelasticity. Error analysis based on our experimental data reveals that our technique can measure stress-optical coefficients with an accuracy down to 10^−12^ Pa^−1^ (Supplementary Information Section IX), qualifying it as a highly sensitive method for characterizing photoelasticity in thin film optical devices.

## Conclusions

In conclusion, we have demonstrated the first single-mode stretchable integrated photonic devices. The stretchable photonics platform has been implemented in two material systems: high-index chalcogenide glasses and low-index epoxy polymers, both monolithically integrated on PDMS elastomer substrates. We have further realized low-loss optical coupling and seamless co-integration of the two systems on a common substrate platform. Our unique approach allows significant design flexibility to garner performance gains from both high-index-contrast and low-index-contrast photonic designs.

Our micro-mechanical engineering strategies towards enhancing stretching capabilities involve local substrate stiffening to protect functional photonic components, and an Euler spiral based interconnecting waveguide design which effectively suppresses both local strain and radiative optical loss. Photonic devices fabricated following this configuration exhibit remarkable mechanical ruggedness and can readily withstand 3000 stretching cycles at 41% nominal strain without compromising their structural integrity and optical performance. Our design approach is also non-material-specific and can be generically applied to transform intrinsically rigid or brittle materials into a highly stretchable and optically functional form.

Finally, we have also derived and experimentally verified a rigorous theory enabling predictive modeling of strain-optical coupling behavior in photonic devices for the first time. The theory is widely applicable to soft materials and mechanically compliant device architectures in photonics, which are becoming increasingly prevalent as integrated photonics continues to penetrate emerging applications such as biomedicine, sensing, and short-reach communications.

## Author contributions

LL and HL conceived the device designs and carried out device fabrication and testing. SQ performed mechanical modeling and analysis. YH, CR and LV assisted in device testing. JL and JM contributed to thin film deposition. AY synthesized and characterized the glass materials. JH, TG, NL and KR supervised and coordinated the project. All authors contributed to writing the paper.
