# Supplementary material for: Monolithically integrated stretchable photonics
Source: Light Sci Appl. 2018 Feb 9;7:17138–. doi: 10.1038/lsa.2017.138 (PMC6060064; doi:10.1038/lsa.2017.138)
Supplement: Supplementary Information [file lsa2017138x1.docx]

**Supplementary Information for**

**Monolithically Integrated Stretchable Photonics**

**Lan Li^1^**^†,*^**, Hongtao Lin^1†^, Shutao Qiao^2^**^†^**, Yi-Zhong Huang^1,3^, Jun-Ying Li^1,4^, Jérôme Michon^1^, Tian Gu^1^, Carlos Alosno-Ramos^5^, Laurent Vivien^5^, Anupama Yadav^6^, Kathleen Richardson^6^, Nanshu Lu^2^, Juejun Hu^1,*^**

*^1^Department of Materials Science & Engineering, Massachusetts Institute of Technology, Cambridge,* *Massachusetts, USA*

*^2^Department of Aerospace Engineering and Engineering Mechanics, University of Texas at Austin, Austin, Texas, USA*

*^3^Department of Electronic Engineering, Xiamen University, Xiamen, China*

*^4^Key Laboratory of Optoelectronic Technology & System, Education Ministry of China, Chongqing University, Chongqing, China*

*^5^Centre for Nanoscience and Nanotechnology, CNRS, Univ. Paris-Sud, Universite Paris-Saclay, C2N – Orsay, Orsay cedex, France*

*^6^The College of Optics & Photonics, University of Central Florida, Orlando, Florida, USA*

*† These authors contributed equally to this work.*

***[*lanli1@mit.edu*](mailto:lanli1@mit.edu)*,* [*hujuejun@mit.edu*](mailto:hujuejun@mit.edu)

In this Supplementary Information, we provide further details on device modeling, fabrication, and characterization results. This Supplementary Information comprises the following Sections:

1. Direct photonic device fabrication on PDMS: the impact of SU-8 buffer layer
2. Adiabatic coupling between low-index-contrast and high-index-contrast optics
3. Optical loss comparison in circular and Euler spiral waveguide bends
4. Structural integrity of devices after 3,000 stretching cycles
5. Derivation of strain-optical coupling theory in guided wave photonic devices
6. Coupling of shear stresses to optical modes
7. Extracting *C_R_* and *C_θ_* from calibration sample measurements
8. Quantification of strain-optical coupling contributions from geometric and photoelastic terms
9. Error analysis of stress-optical coefficient measurement

**Section I – Direct photonic device fabrication on PDMS: the impact of SU-8 buffer layer**

Direct deposition and lithographic patterning of Ge_23_Sb_7_S_70_ chalcogenide glass were attempted on PDMS films coated on a silicon handler wafer. While we were able to obtain crack-free Ge_23_Sb_7_S_70_ films on PDMS via thermal evaporation (as the substrates were held near room temperature during deposition), subsequent lithographic processing resulted in extensive cracking throughout the samples (Figs. S1a-S1c). This is presumably caused by the large CTE mismatch between Ge_23_Sb_7_S_70_ glass (17.1 ppm °C^-1^) and PDMS (310 ppm °C^-1^), which leads to thermal stress accumulation during resist baking steps.

The cracking issue is resolved by inserting a cured SU-8 layer between the Ge_23_Sb_7_S_70_ film and the PDMS substrate. Once cured, SU-8 becomes a robust thermosetting polymer with a tensile strength of 60 MPa[^1^](#_ENREF_1). Since the maximum thermal stress in the film is estimated to be less than 0.4 MPa based on our processing temperature, the SU-8 layer can maintain its structural integrity and inhibit crack initiation. SU-8 also has an intermediate CTE of 52 ppm °C^-1^, and the reduced CTE mismatch between SU-8 and Ge_23_Sb_7_S_70_ glass effectively prevent damage of the glass film. As shown in Fig. S1d, Ge_23_Sb_7_S_70_ glass structured defined on SU-8/PDMS exhibit excellent pattern fidelity with no sign of cracking.


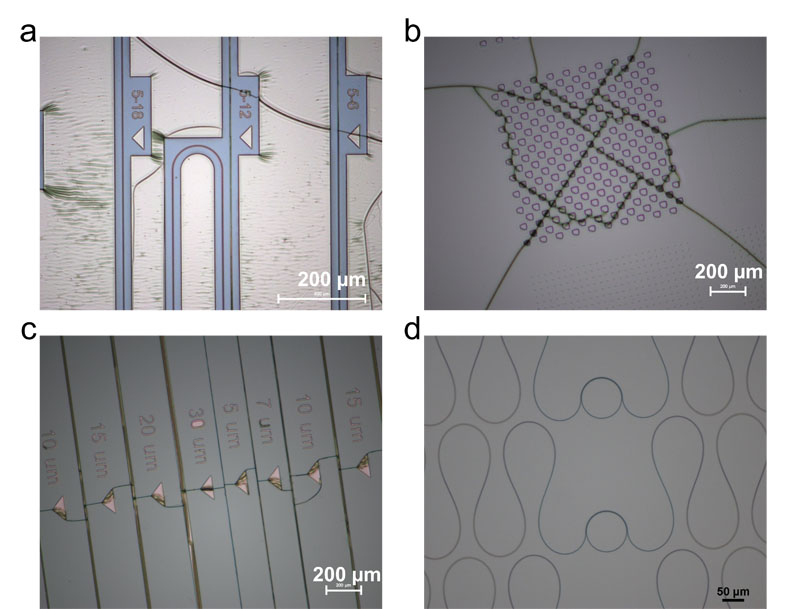


**Figure S1. (a-c) Micrographs of Ge_23_Sb_7_S_70_ glass patterns directly fabricated on PDMS; (d) Ge_23_Sb_7_S_70_ glass waveguides fabricated on SU-8 as a buffer layer on top of PDMS.**

**Section II – Adiabatic coupling between low-index-contrast and high-index-contrast optics**

Low-loss transition between the high-index-contrast ChG/SU-8 waveguides and the low-index-contrast SU-8/PDMS guides is realized via an adiabatic mode transformer. The mode size converter made of Ge_23_Sb_7_S_70_ glass embedded in an SU-8 strip waveguide and assumes an inverse taper geometry. The ChG/SU-8 waveguide and mode transformer design parameters are illustrated in Fig. S2. The same set of geometric parameters were adopted for stretchable devices described throughout this article.

Insertion loss of the mode transformer is measured by taking the difference of optical transmittances (in dB) through the structures depicted in Fig. S2b and Fig. S2a of the same total length. Five pairs of devices with identical configurations were measured, yielding an average insertion loss of (0.4 ± 0.2) dB for the mode transformer.


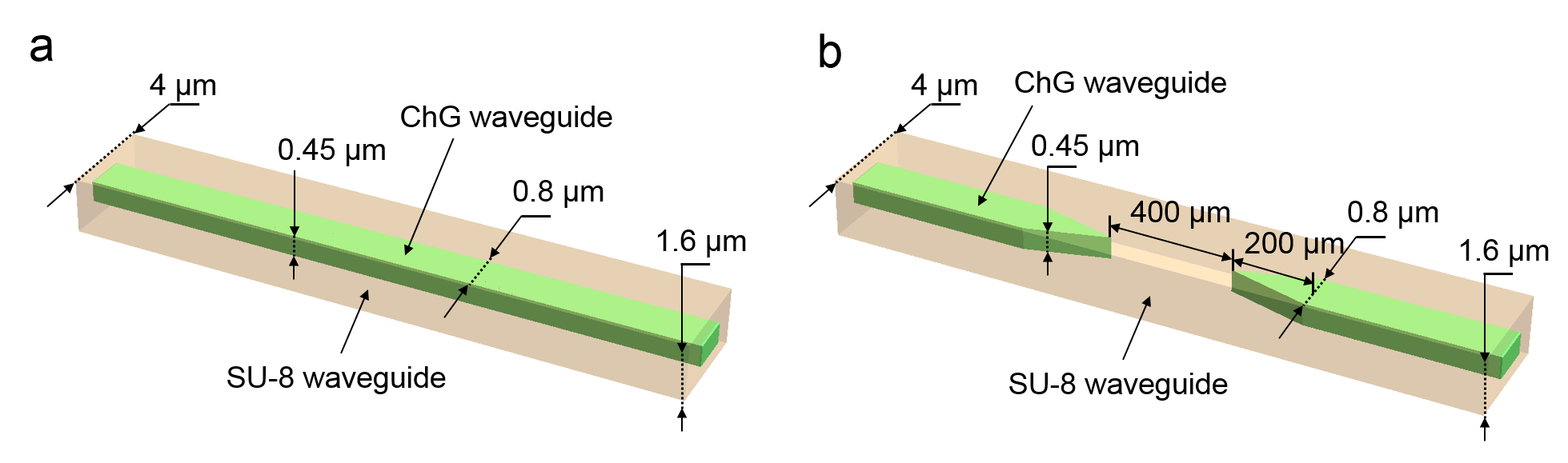


**Figure S2. Waveguide and mode transformer design: (a) a ChG/SU-8 waveguide; and (b) two mode transformers connected back-to-back in series for insertion loss characterization.**

**Section III –** **Optical loss comparison in circular and Euler spiral waveguide bends**


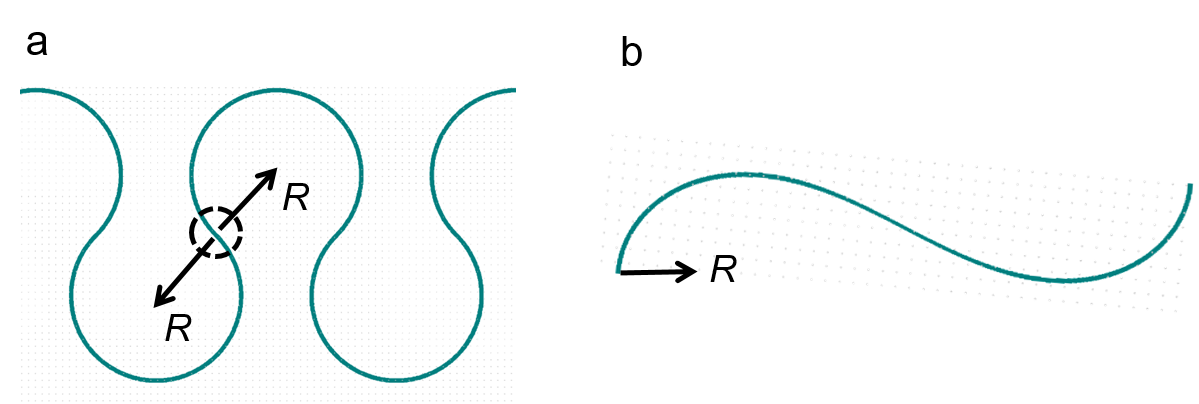


**Figure S3. Two meandering ChG/SU-8 waveguide configurations for stretchable photonics comprising (a) circular segments; and (b) Euler spirals (the graph shows half a period).**

Figures S3a and S3b illustrate the two meandering ChG/SU-8 waveguide configurations under comparison, where *R* denotes the local radius curvature. Propagation losses in the waveguides were measured using a “cut-back” method in serpentine waveguides containing varying numbers of bending segments (repeating units). Table S1 summarizes measured losses in the devices. The much higher propagation loss in the serpentine waveguides consisting of circular segments is attributed to mode mismatch at the abrupt junctions between the circular segments. At 50 μm bending radius, the Euler spiral design has a loss figure similar to that of straight Ge_23_Sb_7_S_70_ glass waveguides (~ 4 dB cm^-1^). We have therefore adopted the Euler spiral structure with 50 μm bending radius to construct our stretchable photonic circuits.

**Table S1. Measured optical propagation losses in serpentine ChG/SU-8 waveguides composed of circular and Euler spiral segments**

|  | Radius *R* (µm) | Loss (dB cm^-1^) |
| --- | --- | --- |
| Circular | 25 | 12.5 |
| Circular | 50 | 7.7 |
| Euler spiral | 25 | 4.5 |
| Euler spiral | 50 | 4.3 |

**Section IV –** **Structural integrity of devices after 3,000 stretching cycles**

Figure S4 presents exemplary optical micrographs of serpentine waveguide structures after 3,000 stretching cycles at 41% nominal strain. Careful visual inspection through optical microscopy did not reveal any sign of cracking or delamination at different locations of the devices. No change in optical transmittance of the waveguides were detected after the repeated stretching cycles within our measurement error. These results attest to the superior mechanical ruggedness of our stretchable optical devices.


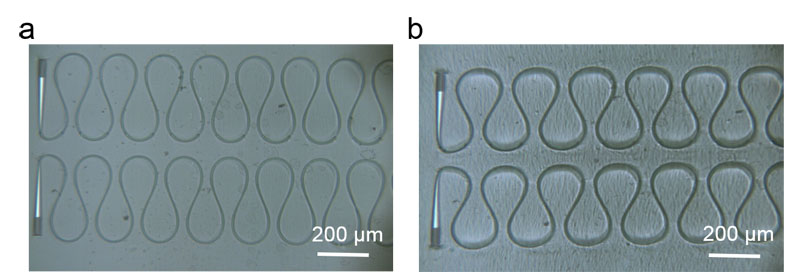


**Figure S4. Optical microscope images of serpentine waveguides after 3,000 stretching cycles at 41% nominal strain (a) in its undeformed state; and (b) at 36% nominal strain. No cracks were visually observed in both cases.**

**Section V – Derivation of strain-optical coupling theory in guided wave photonic devices**

In this section, we seek to derive a generic strain-optical coupling formalism which relates strain/stress fields to optical property changes of guided wave devices. For generality, here we consider spatially non-uniform strain/stress fields, take into account the tensorial nature of strains and stresses, and place no constraints on the geometric configurations of the waveguide structure. The following equations are derived for a travel-wave resonator, although the same analytical approach can be readily applied to other types of guided wave devices.

The resonant condition for a traveling wave resonator can be generically expressed as:

$\int_{L_{\text{tot}}} n_{\text{eff}}\text{d}L=N\lambda_{0}$ (1)

where the integral is performed along the length of the entire resonator. Here *n*_eff_ denotes the modal effective index, *L*_tot_ is the cavity length, *λ*_0_ represents the resonant wavelength in free space, and *N* gives the longitudinal mode order. Linearity of the equation indicates that when a spatially varying perturbation to the optical path length is imposed on the resonator, the overall effect is equivalent to the sum of perturbative effects from each resonator segment d*L*. We therefore start with considering a stress-induced perturbation on one traveling wave resonator segment d*L*. The optical path length change caused by applied stress *σ* on the segment d*L* is:

$\frac{\partial\left(n_{\text{eff}}\text{d}L\right)}{\partial\sigma}\cdot\sigma=\left(n_{\text{eff}}\frac{\partial\varepsilon_{L}}{\partial\sigma}+\frac{\partial n_{\text{eff}}}{\partial\sigma}+\frac{\partial n_{\text{eff}}}{\partial\lambda_{0}}\frac{\partial\lambda_{0}}{\partial\sigma}\right)\cdot\sigma\text{d}L$ (2)

The three terms in the parentheses on the right-hand-side correspond to waveguide length change, stress-induced effective index modification (which include both photoelastic material index change and waveguide cross-sectional geometry deformation), and dispersive effects, respectively. *ε_L_* denotes normal strain along the waveguide segment. The resulting resonant wavelength detuning is given by:

$\int_{L_{\text{tot}}-\text{d}L} \left(n_{\text{eff}}+\frac{\partial n_{\text{eff}}}{\partial\lambda_{0}}\cdot\text{d}\lambda_{0,\text{d}L}\right)\text{d}L+n_{\text{eff}}\text{d}L+\frac{\partial\left(n_{\text{eff}}\text{dL}\right)}{\partial\sigma}\cdot\sigma=N\left(\lambda_{0}+\text{d}\lambda_{0,\text{d}L}\right)$ (3)

The first term on the left-hand-side manifests the dispersive effect imposed on the unperturbed waveguide segments. The integration is carried out along the resonator skipping the d*L* segment. Eq. 3 simplifies to:

$\frac{\partial\left(n_{\text{eff}}\text{d}L\right)}{\partial\sigma}\cdot\sigma=\left(N-\int_{L_{\text{tot}}-\text{d}L} \frac{\partial n_{\text{eff}}}{\partial\lambda_{0}}\text{d}L\right)\cdot\text{d}\lambda_{0,\text{d}L}$ (4)

$\Rightarrow\left(N-\int_{L_{\text{tot}}-\text{d}L} \frac{\partial n_{\text{eff}}}{\partial\lambda_{0}}\text{d}L\right)\cdot\text{d}\lambda_{0,\text{d}L}=\left(n_{\text{eff}}\frac{\partial\varepsilon_{L}}{\partial\sigma}+\frac{\partial n_{\text{eff}}}{\partial\sigma}+\frac{\partial n_{\text{eff}}}{\partial\lambda_{0}}\frac{\partial\lambda_{0}}{\partial\sigma}\right)\cdot\sigma\text{d}L$ (5)

$\Rightarrow\left(N-\int_{L_{\text{tot}}} \frac{\partial n_{\text{eff}}}{\partial\lambda_{0}}\text{d}L+\frac{\partial n_{\text{eff}}}{\partial\lambda_{0}}\text{d}L\right)\cdot\text{d}\lambda_{0,\text{d}L}=\left(n_{\text{eff}}\frac{\partial\varepsilon_{L}}{\partial\sigma}+\frac{\partial n_{\text{eff}}}{\partial\sigma}+\frac{\partial n_{\text{eff}}}{\partial\lambda_{0}}\frac{\partial\lambda_{0}}{\partial\sigma}\right)\cdot\sigma\text{d}L$ (6)

Assuming that the unperturbed travel wave resonator is made up of waveguides with a uniform cross-section, we have:

$n_{\text{eff}}L_{\text{tot}}=N\lambda_{0}$ (7)

Combining Eqs. 6 and 7 yields:

$\left(\frac{n_{\text{eff}}L_{\text{tot}}}{\lambda_{0}}-\frac{\partial n_{\text{eff}}}{\partial\lambda_{0}}L_{\text{tot}}+\frac{\partial n_{\text{eff}}}{\partial\lambda_{0}}\text{d}L\right)\cdot\text{d}\lambda_{0,\text{d}L}=\left(n_{\text{eff}}\frac{\partial\varepsilon_{L}}{\partial\sigma}+\frac{\partial n_{\text{eff}}}{\partial\sigma}+\frac{\partial n_{\text{eff}}}{\partial\lambda_{0}}\frac{\partial\lambda_{0}}{\partial\sigma}\right)\cdot\sigma\text{d}L$ (8)

Since the stress-induced effects can be treated as a high-order perturbation, we also have:

$\frac{\partial\lambda_{0}}{\partial\sigma}=\frac{\text{d}\lambda_{0,\text{d}L}}{\sigma}$ (9)

Eqs. 8 and 9 lead to:

$\frac{L_{\text{tot}}}{\lambda_{0}}\left(n_{\text{eff}}-\lambda_{0}\frac{\partial n_{\text{eff}}}{\partial\lambda_{0}}\right)\cdot\text{d}\lambda_{0,\text{d}L}=\left(n_{\text{eff}}\frac{\partial\varepsilon_{L}}{\partial\sigma}+\frac{\partial n_{\text{eff}}}{\partial\sigma}\right)\cdot\sigma\text{d}L$ (10)

By definition, the waveguide group index is:

$n_{\text{g}}=n_{\text{eff}}-\lambda_{0}\frac{\partial n_{\text{eff}}}{\partial\lambda_{0}}$ (11)

Eq. 10 thereby simplifies to:

$\text{d}\lambda_{0,\text{d}L}=\frac{\lambda_{0}}{L_{\text{tot}}n_{\text{g}}}\left(n_{\text{eff}}\frac{\partial\varepsilon_{L}}{\partial\sigma}+\frac{\partial n_{\text{eff}}}{\partial\sigma}\right)\cdot\sigma\text{d}L$ (12)

We note that the wavelength shift scales linearly with both the magnitude of the perturbation (*σ*) and the segment length d*L*, consistent with the linear nature of the resonant condition Eq. 1. Consequently, the total resonant wavelength shift when a stress field *σ *(*L*) is exerted on the traveling wave resonator can be obtained through a line integration along the resonator perimeter:

$\text{d}\lambda_{0}=\int_{L} \text{d}\lambda_{0,\text{d}L}=\frac{\lambda_{0}}{L_{\text{tot}}n_{\text{g}}}\int_{L} \left(n_{\text{eff}}\frac{\partial\varepsilon_{L}}{\partial\sigma}+\frac{\partial n_{\text{eff}}}{\partial\sigma}\right)\cdot\sigma\left(L\right)\text{d}L$ (13)

Here the spatial variation of *σ* is accounted for through the dependence of *σ* on *L* (denoting the location along the resonator). In the general case where multiple stress components co-exist, the left-hand-side should be the summation of individual stress component effects.

As an example, let’s consider photonic devices fabricated on a thin membrane (as is the case for most flexible photonic devices as well as our stretchable devices). The geometric configuration supports in-plane biaxial stresses. For convenience, we employ a polar coordinate whose origin is located at the center of the micro-ring resonator (Fig. 3c). The two in-plane stress components are labeled as *σ_R_* (normal stress along the in-plane transverse direction to the waveguide) and *σ_θ_* (normal stress along the longitudinal direction along the waveguide). For the stretchable micro-ring resonator, Eq. 13 becomes:

$\text{d}\lambda_{0}=\frac{\lambda_{0}}{L_{\text{tot}}n_{\text{g}}}\int_{0}^{2\pi} \left[C_{R}\cdot\sigma_{R}\left(L\right)+C_{\theta}\cdot\sigma_{\theta}\left(L\right)\right]\cdot R\text{d}\theta$ (14)

where *R* denotes the resonator radius in the absence of perturbation and the two stress-optical coupling constants are defined as follows:

$C_{R}=n_{\text{eff}}\frac{\partial\varepsilon_{L}}{\partial\sigma_{R}}+\frac{\partial n_{\text{eff}}}{\partial\sigma_{R}}$ (15)

and

$C_{\theta}=n_{\text{eff}}\frac{\partial\varepsilon_{L}}{\partial\sigma_{\theta}}+\frac{\partial n_{\text{eff}}}{\partial\sigma_{\theta}}$ (16)

We note that first-principle calculations of the two stress-optical coupling constants can be challenging, since the photoelastic constants of the constituent materials are not always known. Nevertheless, these constants can be experimentally measured using a calibration sample. The calibration sample contains two sets of waveguide Fabry-Perot (F-P) cavities oriented in orthogonal directions, where each set of the F-P cavities contains a series of devices with identical Bragg mirror designs but varying cavity lengths *L*_tot_. The cavities are formed by engraving two Bragg grating reflectors on waveguides. The waveguide dimensions of the calibration sample are chosen such that they match those of the stretchable micro-ring resonator sample. The F-P cavity’s resonant condition is given by:

(17)

where *ϕ*_m_ is the phase delay imparted upon Bragg mirror reflection in radians. The calibration sample is uniaxially bent (or stretched) along two orthogonal directions parallel to the two sets of F-P cavities. The bending (or stretching) action exerts in-plane bi-axial stresses on the F-P cavities along longitudinal and transverse directions with respect to the F-P cavity. Unlike the case of the stretchable resonators, stresses in the calibration sample resulting from uniaxial bending or stretching are spatially uniform. The resonance detuning can therefore be deduced from the F-P cavity resonant condition:

(18)

Taking derivative with respect to a stress component *σ*:

(19)

(20)

(21)

(22)

Eqs. 19-22 apply to both longitudinal and transverse stress components, and *C* represents the stress-optical coupling constant defined in Eqs. 15 and 16. When the calibration sample undergo bending or stretching, Eq. 22 yields the resonant wavelength shifts of the two orthogonal sets F-P cavity devices (labeled as set A and set B) as:

(23)

(24)

Since the stress-optical coupling constants *C*’s are identical for F-P cavities of different lengths and define *γ* = *σ*_2_/*σ*_1_, the equations reduce to:

(25)

(26)

The terms marked with red color in Eqs. 25 and 26 are associated with contribution from the Bragg reflectors. Plotting the resonance shifts measured from F-P cavities of different lengths as a function of 1/*L*_tot_ allows subtraction of the Bragg reflector contribution:

$\frac{\text{d}\lambda_{0,\text{A}}}{\sigma_{1}}\left(\frac{1}{L_{\text{tot}}}\to0\right)=\frac{\lambda_{0,\text{A}}}{n_{\text{g}}}\cdot C_{\theta}+\frac{\lambda_{0,\text{A}}}{n_{\text{g}}}\cdot\gamma C_{R}$ (27)

$\frac{d\lambda_{0,\text{B}}}{\sigma_{1}}\left(\frac{1}{L_{tot}}\to0\right)=\frac{\lambda_{0,\text{B}}}{n_{g}}\cdot C_{R}+\frac{\lambda_{0,\text{B}}}{n_{g}}\cdot\gamma C_{\theta}$ (28)

Solving the two linear equations allows extraction of the two stress-optical coupling constants *C_R_* and *C_θ_*. Knowledge of the stress distribution from finite element simulations thereby enables quantitative prediction of stress-induced resonant wavelength shift in a stretchable resonator by integrating Eq. 14. As an example, Supplementary Section VII presents the experimental data and detailed protocols for *C_R_* and *C_θ_* parameterization in our stretchable ChG/SU-8 devices.

**Section VI – Coupling of shear stresses to optical modes**

In this section, we prove that shear stresses have negligible influence of the optical modal properties of guided wave devices. Shear stresses can modify both the dielectric tensor of waveguide’s constituent materials and the waveguide geometry. In the following we consider both effects. For the sake of convenience, we label the light propagation direction in the waveguide as *x*, and the out-of-plane direction as *z*.

In isotropic materials such as glasses and polymers, the photoelasticity tensor can be generically written as:

$p=\left[\begin{matrix} p_{11} & p_{12} & p_{12} & 0 & 0 & 0 \\ p_{12} & p_{11} & p_{12} & 0 & 0 & 0 \\ p_{12} & p_{12} & p_{11} & 0 & 0 & 0 \\ 0 & 0 & 0 & p_{44} & 0 & 0 \\ 0 & 0 & 0 & 0 & p_{44} & 0 \\ 0 & 0 & 0 & 0 & 0 & p_{44} \end{matrix}\right]$ (29)

Here we follow the formalism by Chen *et al.*[^2^](#_ENREF_2) and define the photoelasticity tensor through:

$\Delta B=p\cdot S$ (30)

In Eq. 30, Δ***B*** is the strain-induced modification of the relative dielectric impermeability tensor ***B*** (defined as 1/***ε* _r_** where ***ε* _r_** is the relative permittivity tensor), and ***S*** denotes the strain tensor. Both tensors have six independent elements and thus ***p*** is a fourth order tensor represented by a 6 × 6 matrix.

If the applied strain is sufficiently small such that the material is Hookean and that the change of the dielectric constant is small, we have:

$\Delta\varepsilon_{\text{r}}=P\cdot T$ (31)

where Δ***ε*_r_** gives the relative permittivity change (which is a second order tensor) and ***T*** represents the stress tensor. The fourth order tensor ***P*** is given by:

$P=-\frac{\varepsilon_{\text{r}}^{2}}{Y}\left[\begin{matrix} p_{11}-2\sigma p_{12} & p_{12}-\sigma\left(p_{11}+p_{12}\right) & p_{12}-\sigma\left(p_{11}+p_{12}\right) & 0 & 0 & 0 \\ p_{12}-\sigma\left(p_{11}+p_{12}\right) & p_{11}-2\sigma p_{12} & p_{12}-\sigma\left(p_{11}+p_{12}\right) & 0 & 0 & 0 \\ p_{12}-\sigma\left(p_{11}+p_{12}\right) & p_{12}-\sigma\left(p_{11}+p_{12}\right) & p_{11}-2\sigma p_{12} & 0 & 0 & 0 \\ 0 & 0 & 0 & \frac{p_{44}Y}{\mu} & 0 & 0 \\ 0 & 0 & 0 & 0 & \frac{p_{44}Y}{\mu} & 0 \\ 0 & 0 & 0 & 0 & 0 & \frac{p_{44}Y}{\mu} \end{matrix}\right]$ (32)

In Eq. 32, the unbolded *ε* _r_ is the relative permittivity (a scalar number for isotropic solids), *Y* and *μ* are the Young’ modulus and shear modulus, respectively, and *σ* denotes the Poisson ratio.

Since the change of the dielectric constant is small, optical mode variation due to the stress-induced permittivity modification can be calculated using the classical perturbation theory. For a waveguide device, the propagation constant change Δ*β* is expressed as[^3^](#_ENREF_3):

$\Delta\beta=\frac{\omega\varepsilon_{0}}{2}\cdot\frac{\iint\text{d}^{2}r\cdot\left(\Delta\varepsilon_{\text{r}}\overrightarrow{E}\right)\cdot\overrightarrow{E}*}{\iint\text{d}^{2}r\cdot\mathrm{Re}\left(\overrightarrow{E}\times\overrightarrow{H}*\right)\cdot\hat{x}}$ (33)

where $\overrightarrow{E}$ and $\overrightarrow{H}$ are the unperturbed mode, *ω* is the angular frequency of light, and *ε* _0_ gives the vacuum permittivity.

Eqs. 31 and 32 specify that shear stresses only affect the off-diagonal terms in the permittivity tensor. Based on Eq. 33, the perturbative effects scales with the term $\left(\Delta\varepsilon_{\text{r}}E\right)\cdot E*$, which vanishes for the off-diagonal permittivity tensor terms.

Next, we investigate the geometric effects imposed by shear stresses. In a thin-membrane flexible photonic device with free surfaces, only the in-plane shear stress component *T_xy_* is present. Modal perturbation resulting from the shear deformation caused by *T_xy_* is most conveniently evaluated by invoking the perturbation theory involving shifting material boundaries[^4^](#_ENREF_4):

$\Delta\omega=-\frac{\omega}{2}\cdot\frac{\iint\text{d}^{2}r\cdot\left[\left(\varepsilon_{1}-\varepsilon_{2}\right)\left|E_{//}\left(r\right)\right|^{2}-\left(\frac{1}{\varepsilon_{1}}-\frac{1}{\varepsilon_{2}}\right)\left|\varepsilon E_{\perp}\left(r\right)\right|^{2}\right]\cdot\Delta h}{\iiint\text{d}^{3}r\cdot\varepsilon\left(r\right)\left|E\left(r\right)\right|^{2}}$ (34)

where *ε* _1_ and *ε* _2_ represent the permittivity on two sides of the shifting boundary, $E_{//}$ and $E_{\perp}$ are the modal electric field parallel and perpendicular to the boundary respectively, and Δ*h* denotes displacement of the boundary. In a device exhibiting mirror symmetry with respect to the *x* or *y* axis, the optical mode must also be symmetric or anti-symmetric about the same axis. The terms containing |*E|*^2^ thus obey mirror symmetry. Since the shear stress *T_xy_* generates an anti-symmetric displacement field about the axis of symmetry, it is straightforward to deduce from Eq. 34 that perturbative effects due to shifted boundaries on two sides of the symmetric device structure cancel out each other. We therefore conclude that shear stresses have a negligible contribution to optical mode perturbation.

**Section VII – Extracting C*_R_* and C*_θ_* from calibration sample measurements**

A calibration sample containing two set of waveguide F-P cavities with orthogonal orientations were fabricated on flexible substrates following protocols employed by our previously demonstrated foldable photonic devices[^5^](#_ENREF_5). Each set includes four devices with varying cavity length *L*_tot_. The sample was then bent to different radii while the resonant wavelengths of the F-P cavities were monitored (Fig. S7a). Orientations of the two sets of devices (labeled as A and B) with respect to the bending direction are illustrated in Fig. 4a. As an example, Figures S5 presents the evolution of transmission spectra of devices when they were bent to different radii, and Fig. S6 summarizes the F-P cavity resonance peak detuning for both sets of devices as functions of local stresses exerted on the devices during bending, which were modeled using FEM simulations based on given bending radii of the sample.


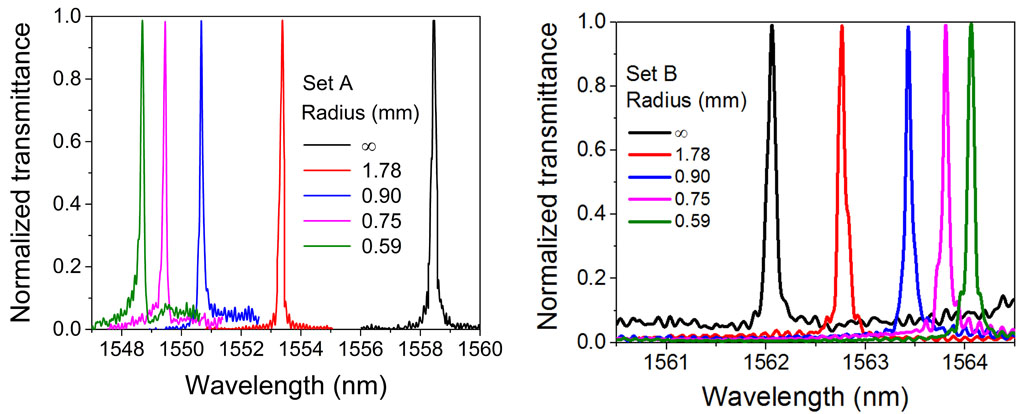


**Figure S5. Exemplary transmission spectra of F-P cavity devices in (left) set A and (right) set B, showing resonance spectral detuning due to bending deformation.**


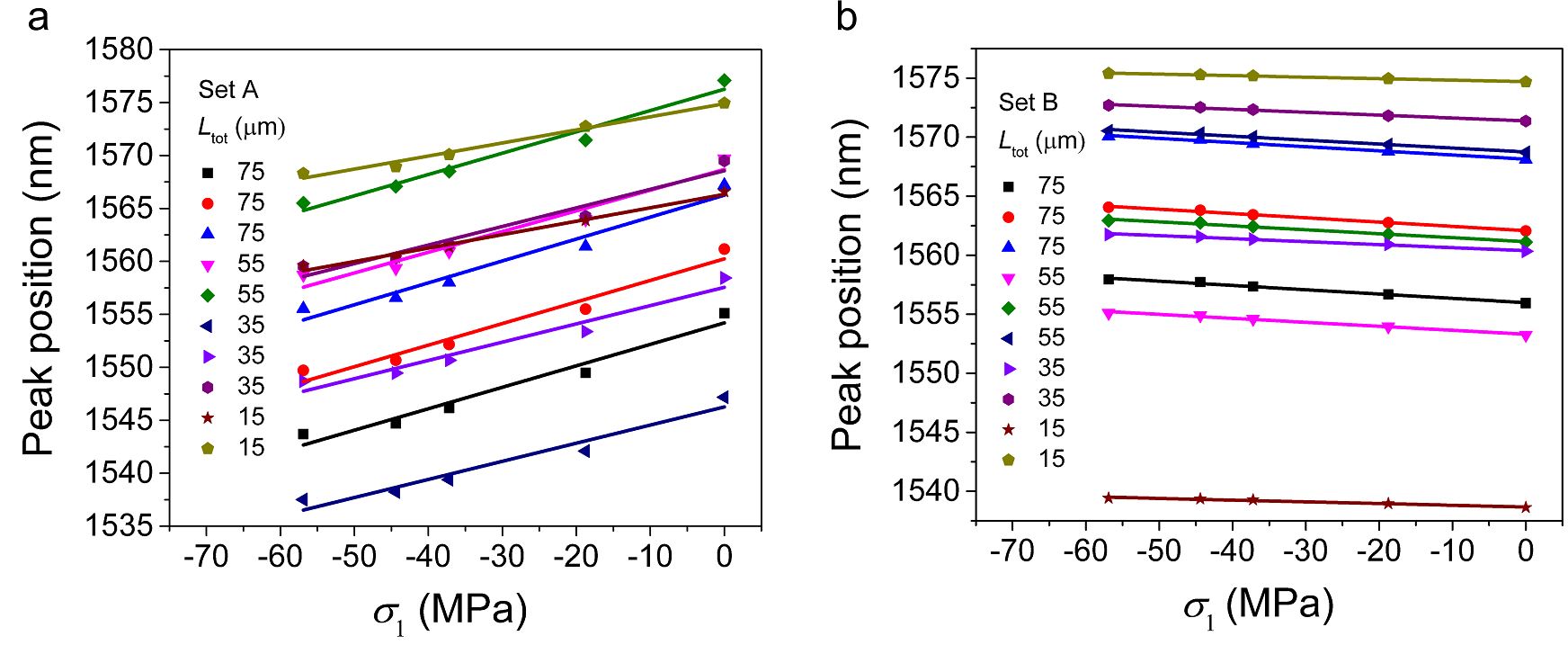


**Figure S6. Resonant peak positions as functions of local stress for device (a) set A and (b) set B.**

In the next step, the slopes d*λ*/*σ*_1_ were computed from Fig. S6 for all the curves, averaged for each cavity length *L*_tot_, and plotted against 1/*L*_tot_ for each device set as shown in Fig. S7. Intercepts of the lines in Fig. S7b with the vertical axis 1/*L*_tot_ = 0 correspond to $\frac{\text{d}\lambda_{0,\text{A}}}{\sigma_{1}}\left(\frac{1}{L_{\text{tot}}}\to0\right)$ and $\frac{\text{d}\lambda_{0,\text{B}}}{\sigma_{1}}\left(\frac{1}{L_{\text{tot}}}\to0\right)$. *C_R_* and *C_θ_* are then solved by combining Eqs. 27 and 28 in Supplementary Section V. Our measurements yield *C_R_* = (1.32 ± 0.08) × 10^-10^ Pa^-1^ and *C_θ_* = (3.42 ± 0.08) × 10^-10^ Pa^-1^. The error bars associated with these stress-optical coupling constants are quantitatively evaluated in Supplementary Section IX.


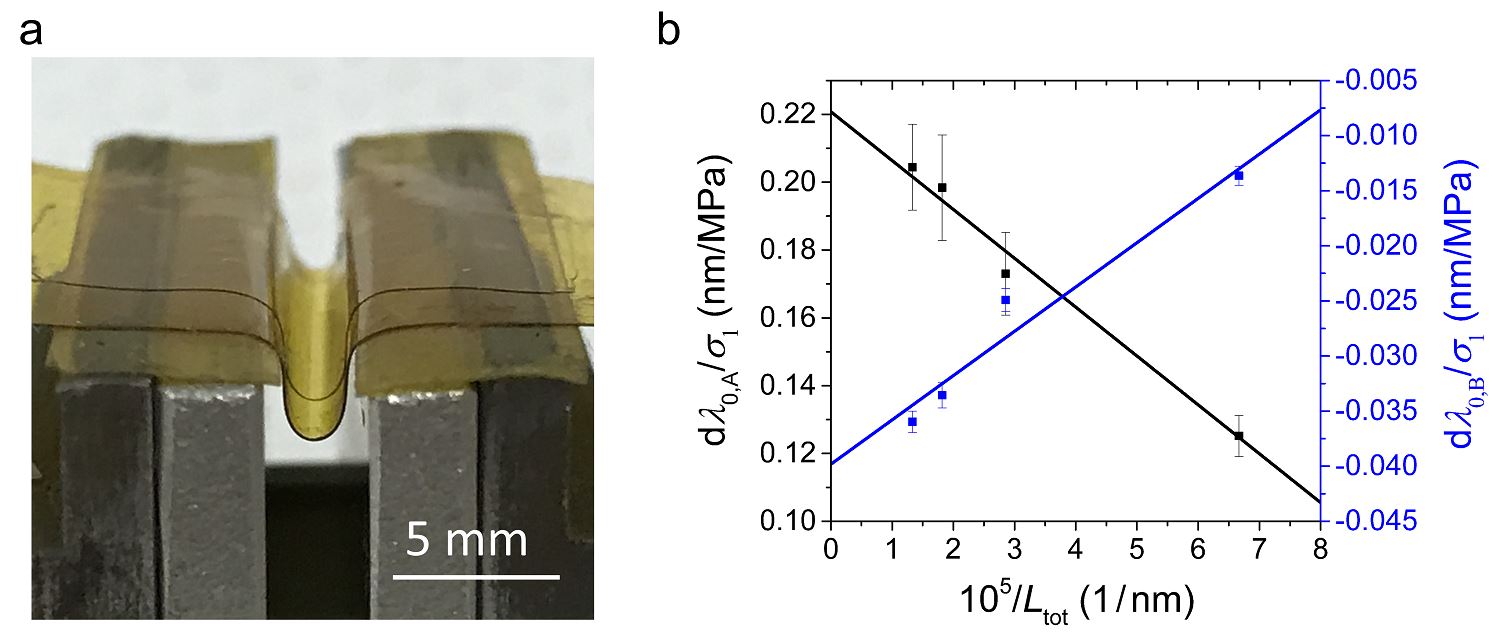


**Figure S7. (a) Photo of a calibration sample under test; (b) d*λ*_0_/*σ*_1_ obtained from Fig. S6 for device set A (black) and B (blue). The dots are data calculated from Fig. S6 and the lines are linear fits.**

**Section VIII – Quantification of strain-optical coupling contributions from geometric and photoelastic terms**

Here we consider two waveguide configurations under stress as sketched in the insets of Figures 4f and 4g. Since in-plane normal stresses are the only stress components affecting the optical modal characteristics according to Supplementary Section VI, linear combinations of the two cases cover all possible scenarios of stressed flexible membrane waveguide devices.

For the axially stressed case shown in Fig. 4f inset, the stress-induced resonance drift is:

$\text{d}\lambda_{0}=\frac{\lambda_{0}}{n_{\text{g}}}\cdot C_{\theta}\sigma$ (35)

Similarly, the resonance shift when stress is applied in the transverse direction (Fig. 4g) is:

$\text{d}\lambda_{0}=\frac{\lambda_{0}}{n_{\text{g}}}\cdot C_{R}\sigma$ (36)

For a given stress magnitude, the device geometric deformation (length change and waveguide cross-section modification) was calculated through FEM mechanical modeling. The waveguide cross-section modifications were then fed into a waveguide mode solver (MODE Solutions, Lumerical Solutions, Inc.) to compute the effective index perturbation. Finally, the photoelastic contributions were quantified by subtracting the two geometric terms from the total resonance shift given by Eqs. 35 and 36.

Table S2 compiles the calculation results. In the table, the stress value refers to stress in the SU-8 cladding. Since the final results are normalized, we fix the stress at 1 MPa for this calculation. Figures 4f and 4g were plotted using the normalized data in the last column.

**Table S2. Quantifying contributions from geometric and photoelastic terms to resonance shift**

|  | Strain-Optical Coupling Mechanism | Contribution to resonance shift (at 1 MPa stress) | Normalized contribution |
| --- | --- | --- | --- |
| Set A | Total | 0.241 | 1 |
|  | Cross-section deformation | -0.056 | -0.23 |
|  | Length change | 0.592 | 2.45 |
|  | Photoelasticity | -0.295 | -1.22 |
| Set B | Total | -0.093 | -1 |
|  | Cross-section deformation | 0.029 | 0.31 |
|  | Length change | -0.050 | -0.53 |
|  | Photoelasticity | -0.072 | -0.78 |

**Section IX – Error analysis of stress-optical coefficient measurement**

This section discusses how the errors of initial experiment results propagate to the final calculated stress-optical coupling coefficients. Through solving the two linear equations Eq. 27 and Eq. 28, the stress-optic coupling constants *C_θ_* and *C_R_* can be expressed as:

$\frac{\lambda_{0}}{n_{\text{g}}}C_{\theta}=\frac{\gamma\frac{\text{d}\lambda_{\text{0,B}}}{\sigma_{1}}\left(\frac{1}{L_{\text{tot}}}\to0\right)-\frac{\text{d}\lambda_{\text{0,A}}}{\sigma_{1}}\left(\frac{1}{L_{\text{tot}}}\to0\right)}{\gamma^{2}-1}$ (37)

$\frac{\lambda_{0}}{n_{\text{g}}}C_{R}=\frac{\gamma\frac{\text{d}\lambda_{\text{0,A}}}{\sigma_{1}}\left(\frac{1}{L_{\text{tot}}}\to0\right)-\frac{\text{d}\lambda_{\text{0,B}}}{\sigma_{1}}\left(\frac{1}{L_{\text{tot}}}\to0\right)}{\gamma^{2}-1}$ (38)

According to the standard addition and subtraction rules of values with error, the uncertainty (standard deviation (S.D.)) of *C_θ_* and *C_R_* are:

$S_{\theta}=S_{R}=\sqrt{S_{\text{A}}^{2}+S_{\text{B}}^{2}}$ (39)

where $S_{\theta},\text{ }S_{R},\text{ }S_{\text{A}}$, and $S_{\text{B}}$ are the S.D. of $C_{\theta},\text{ }C_{R},\text{ }\frac{\text{d}\lambda_{\text{0,A}}}{\sigma_{1}}\left(\frac{1}{L_{\text{tot}}}\to0\right)$, and $\frac{\text{d}\lambda_{\text{0,B}}}{\sigma_{1}}\left(\frac{1}{L_{\text{tot}}}\to0\right)$, respectively. $\frac{\text{d}\lambda_{\text{0,A}}}{\sigma_{1}}\left(\frac{1}{L_{\text{tot}}}\to0\right)$ and $\frac{\text{d}\lambda_{\text{0,B}}}{\sigma_{1}}\left(\frac{1}{L_{\text{tot}}}\to0\right)$ are the intercepts of the fitted lines of$\frac{\text{d}\lambda_{\text{0,A}}}{\sigma_{1}} vs. \frac{1}{L} , \frac{\text{d}\lambda_{\text{0,B}}}{\sigma_{1}} vs. \frac{1}{L}$ shown in Fig. S7b, in which the value and error of each data point d*λ*/*σ*_1_ was calculated from the combination of multiple averaged linear fitting slopes and their errors are shown in Fig. S6.

To understand how the error propagates, first let’s briefly review the standard deviations of line slope and intercept in a linear fitting. Assuming that we have a set of data points whose coordinates are given by *x*_1_, *x*_2_,…, *x_n_* and *y*_1_, *y*_2_,…, *y_n_*, and the *x*’s and *y*’s have a best linear fit expressed as *y* = *kx* + *m*, the fitting error (S.D.) of slope *k* and intercept *m* can be evaluated via:

$S_{k}=S\sqrt{\frac{n}{\left(n\sum x_{i}^{2}\right)-\left(\sum x_{i}\right)^{2}}}$ (40)

$S_{m}=S\sqrt{\frac{\sum x_{i}^{2}}{\left(n\sum x_{i}^{2}\right)-\left(\sum x_{i}\right)^{2}}}$ (41)

$S=\sqrt{\frac{1}{n-2}\sum_{i=1}^{n} \left[y_{i}-\left(m+kx_{i}\right)\right]^{2}}$ (42)

However, if we want to calculate the fitting parameters (slopes and intercepts) and their errors with more than one set of data points, the following equation should be used to combine multiple averaged data points and their errors.

$S_{\text{ab}}=\sqrt{\frac{N_{\text{a}}}{N}S_{\text{a}}^{2}+\frac{N_{\text{b}}}{N}S_{\text{b}}^{2}+\frac{n_{\text{a}}n_{\text{b}}}{nN}(\overline{a}-\overline{b})^{2}}$, where (43)

$n=n_{\text{a}}+n_{\text{b}}$ (44)

$N=n(n-1)$ (45)

$N_{\text{a}}=n_{\text{a}}(n_{\text{a}}-1)$ (46)

$N_{\text{b}}=n_{\text{b}}(n_{\text{b}}-1)$ (47)

Here *S*_a_, *S*_b_, and *S*_ab_ are the standard deviations of data set a, data set b, and the combined data set of a and b; *n*_a_ and *n*_b_ are the number of data points in data sets a and b; and $\overline{a} and \overline{b}$ are the average value of data sets a and b. Repeat the above protocols to obtain S.D. for three or more combined data sets if needed.

To analyze the error propagation in our measurement, we have used the linear fitting function in the Origin software to directly obtain the value and error of fitted slopes d*λ*/*σ*_1_ for each data set in Fig. S6. The details numbers are summarized in Table S3.

**Table S3. Summary of the values and errors of fitted slopes d*λ*/*σ*_1_ in Fig. S6**

| Set A | | | Set B | | |
| --- | --- | --- | --- | --- | --- |
| *L* (μm) | **d*λ*/*σ*_1_**  **(nm MPa^-1^)** | **S.D.**  **(nm MPa^-1^)** | ***L* (μm)** | **d*λ*/*σ*_1_**  **(nm MPa^-1^)** | **S.D.**  **(nm MPa^-1^)** |
| 75 | 0.20261 | 0.02322 | 75 | -0.03647 | 0.00203 |
| 75 | 0.20371 | 0.02379 | 75 | -0.03628 | 0.00186 |
| 75 | 0.20691 | 0.02411 | 75 | -0.03519 | 0.00146 |
| 55 | 0.19546 | 0.02625 | 55 | -0.03369 | 0.00221 |
| 55 | 0.20131 | 0.0199 | 55 | -0.03353 | 0.00224 |
| 35 | 0.17111 | 0.02305 | 55 | -0.03344 | 0.00205 |
| 35 | 0.17249 | 0.02231 | 35 | -0.02543 | 0.00165 |
| 35 | 0.17542 | 0.02302 | 35 | -0.02438 | 0.00139 |
| 15 | 0.1268 | 0.00915 | 15 | -0.01454 | 0.00157 |
| 15 | 0.12347 | 0.00895 | 15 | -0.01275 | 0.00065 |

To combine the results of multiple samples with the same F-P cavity length, Eqs. 43-47 were used to calculate the final values and errors of d*λ*/*σ*_1_ for samples with different cavity lengths. The calculated numbers as well as the fitted intercepts using those numbers in Origin software (Fig. S7) are tabulated in Table S4.

**Table S4. Summary of the values and errors of fitted slopes d*λ*/*σ*_1_ from combining the results of multiples samples in Fig. S6 as well as the linear fitting results in Fig. S7**

| Set A | | | | |
| --- | --- | --- | --- | --- |
| *L* (μm) | **d*λ*/*σ*_1_ (nm/MPa)** | **S.D.**  **(nm/MPa)** | **Fitted intercept**$\frac{\text{d}\lambda_{\text{0,A}}}{\sigma_{1}}\left(\frac{1}{L_{\text{tot}}}\to0\right)$ |  |
| 75 | 0.20441 | 0.012683 | **Value**  **(nm MPa^-1^)** | **S.D. (S_A_)**  **(nm MPa^-1^)** |
| 55 | 0.198385 | 0.015559 | **0.2208** | **0.00507** |
| 35 | 0.173007 | 0.012194 |  |  |
| 15 | 0.125135 | 0.006059 |  |  |

| Set B | | | | |
| --- | --- | --- | --- | --- |
| *L* (μm) | **d*λ*/*σ*_1_**  **(nm MPa^-1^)** | **S.D.**  **(nm MPa^-1^)** | **Fitted intercept** $\frac{\text{d}\lambda_{\text{0,B}}}{\sigma_{1}}\left(\frac{1}{L_{\text{tot}}}\to0\right)$ |  |
| 75 | -0.03598 | 0.000973 | **Value**  **(nm MPa^-1^)** | **S.D. (S_B_)**  **(nm MPa^-1^)** |
| 55 | -0.03355 | 0.001159 | **-0.03982** | **0.00249** |
| 35 | -0.02491 | 0.001032 |  |  |
| 15 | -0.01365 | 0.000855 |  |  |

By substituting the values of $\frac{\text{d}\lambda_{\text{0,A}}}{\sigma_{1}}\left(\frac{1}{L_{\text{tot}}}\to0\right)$ and $\frac{\text{d}\lambda_{\text{0,B}}}{\sigma_{1}}\left(\frac{1}{L_{\text{tot}}}\to0\right)$ in Table S4 into Eq. 37 and Eq. 38 (where Poisson ratio of SU-8 is taken as 0.22), we get:

$\frac{\lambda}{n_{\text{g}}}C_{\theta}=\text{ }0.241236\pm0.005648$ (nm MPa^-1^) (48)

$\frac{\lambda}{n_{\text{g}}}C_{R}=\text{ }-0.092892\pm0.005648$ (nm MPa^-1^) (49)

$S_{\theta}=S_{R}=\sqrt{S_{\text{A}}^{2}+S_{\text{B}}^{2}}=\sqrt{0.00507^{2}+0.00249^{2}}=0.005648$ (nm MPa^-1^) (50)

In our experiment, group index $n_{\text{g}}$of the ChG/SU-8 waveguide is calculated to be 2.2 from our mode solver and the device test wavelength $\lambda_{0}$ is set around 1550 nm, so the stress-optic coupling coefficients are:

$C_{\theta}=\text{ }\left(3.42\text{ }\pm\text{ }0.08\right)\text{ }\times\text{ }10^{-10}$ (Pa^-1^) (51)

$C_{R}=\text{ (}-1.32\text{ }\pm\text{ }0.08)\text{ }\times\text{ }10^{-10}$ (Pa^-1^) (52)

We note that here the two coefficients are defined with respect to the stresses in SU-8. The actual stresses in the Ge_23_Sb_7_S_70_ chalcogenide glass is about 8 times higher given its larger elastic modulus. Therefore, the measurement accuracy for stress-optic coefficients referenced to stresses in the glass material is about 10^-12^ Pa^-1^.

**References**

1 SU-8 Permanent Photoresists. <http://www.microchem.com/pdf/SU-8-table-of-properties.pdf>*, retrieved on 05/01/2017.*

2 Chen CL. *Foundations for guided-wave optics*. John Wiley & Sons 2006.

3 Kogelnik H. in *Guided-wave optoelectronics*. Springer 1988; 7-88.

4 Johnson SG, Ibanescu M, Skorobogatiy MA, Weisberg O, Joannopoulos JD. Perturbation theory for Maxwell’s equations with shifting material boundaries. *Physical Review E* 2002; **65**: 066611.

5 Li L, Lin HT, Qiao ST, Zou Y, Danto S *et* *al*. Integrated flexible chalcogenide glass photonic devices. *Nat* *Photonics* 2014; **8**: 643-649.
